# Induction of remission in diabetes by lowering blood glucose

**DOI:** 10.3389/fendo.2023.1213954

**Published:** 2023-06-20

**Authors:** Gordon C. Weir, Susan Bonner-Weir

**Affiliations:** Joslin Diabetes Center, Harvard Medical School, Boston, MA, United States

**Keywords:** B-cell, insulin, type 2 diabetes (T2D), type 1 diabetes (T1D), diabetes remission, resistance, ketoacidosis

## Abstract

As diabetes continues to grow as major health problem, there has been great progress in understanding the important role of pancreatic beta-cells in its pathogenesis. Diabetes develops when the normal interplay between insulin secretion and the insulin sensitivity of target tissues is disrupted. With type 2 diabetes (T2D), glucose levels start to rise when beta-cells are unable to meet the demands of insulin resistance. For type 1 diabetes (T1D) glucose levels rise as beta-cells are killed off by autoimmunity. In both cases the increased glucose levels have a toxic effect on beta-cells. This process, called glucose toxicity, has a major inhibitory effect on insulin secretion. This beta-cell dysfunction can be reversed by therapies that reduce glucose levels. Thus, it is becoming increasingly apparent that an opportunity exists to produce a complete or partial remission for T2D, both of which will provide health benefit.

As diabetes continues to grow as a major world health problem, there is increased interest in how revival of dysfunctional beta-cells might result in either complete or partial remissions. In one study (1) a complete remission was defined as an HbA1c of below 5.7% and/or a fasting glucose level below 100 mg/dl without medication. A partial remission was defined as a HbA1c of 5.7-6.5% and/or a fasting glucose level of 100-125 mg/dl. A complete remission can only be obtained in a limited number of patients, but a partial remission in many more. Diabetes develops when insufficient insulin is secreted to meet the requirements of an individual’s insulin sensitivity. We typically think of type 2 diabetes (T2D) as being the result of years of insulin insensitivity (insulin resistance) leading to beta-cell secretory dysfunction, while type 1 diabetes (T1D) results mainly from a failure of insulin secretion due to loss of beta-cells from autoimmunity. With remissions occurring in both types of diabetes, insulin secretion and insulin sensitivity should be thought of as determinants and therapeutic opportunities.

Understanding remission requires knowledge of the basic pathophysiology of diabetes. Diabetes is simply defined by the presence of elevated blood glucose levels that are associated with diabetic complications. Glucose levels rise when the beta-cells of the pancreas are unable to meet insulin demand given whatever degree of insulin sensitivity is present. Insulin secretion is to some extent correlated with beta-cell mass, which is known to be quite variable as determined by measurement of beta-cell mass in autopsy studies. It has not been possible to carry out well controlled studies of the relationship between beta-cell mass and function, but it can be assumed that the correlation exists.

## Interplay between insulin secretion and insulin sensitivity

People without diabetes maintain glucose levels within a very narrow range, between 70-150 mg/dl. This impressive homeostasis occurs despite large differences in insulin sensitivity and insulin secretion. Remarkably, insulin sensitivity can vary more that 10-fold (2). Although insulin sensitivity is of obvious importance, its countless intertwined components have proved difficult to dissect. Multiple organs, notably liver, adipose tissue, muscle, and brain have complex interactions. It is important to appreciate and understand this complexity because there are countless potential therapeutic targets.

In contrast to the complexities of what we call insulin sensitivity beta-cells have a more straight-forward task, which is to control plasma glucose levels by secreting as much insulin as is needed. For the fortunate people without diabetes this works incredibly well. When faced with increased insulin sensitivity due to less food and more physical activity beta-cells reduce their secretion with incredible accuracy, and when confronted with insulin resistance they do what is necessary to maintain the same excellent control.

Beta-cells developed to store large amounts of insulin, a portion of which can be released within minutes to deal with a great variety of eating patterns. Even with a regular lifestyle of three meals per day, the right amount of insulin is released for bacon and eggs at breakfast and pasta at a late dinner. After each feeding, the beta-cell rapidly synthesizes more insulin that can be stored ready to be launched as needed.

## When beta-cells cannot secrete enough insulin glucose toxicity becomes a major problem

In adults, beta-cells have a mass of 1-2 grams which represents about 2% of pancreatic volume and consists of approximately one billion beta-cells contained in one million islets. With obesity, a greater mass of beta-cells is found. However, the term beta-cell functional mass refers to changes in secretion without changes in mass, often due to changes in function. Diabetes develops when beta-cell functional mass is inadequate and becomes problematic because elevated glucose levels are toxic to beta-cells, described by the term glucose toxicity (2, 3). Impairments of insulin secretion even show up when glucose levels are minimally elevated and become much worse as glucose levels climb. Much is written about the potential negative effects of free fatty acids (FFA) on insulin secretion, which are described by the terms lipotoxicity and glucolipotoxicity (4). We have reviewed the data associated with these purported effects of FFAs and agree that negative effects of FFAs can be found with *in vitro* experiments but not convincingly in real-life situations (5). Glucose toxicity, however, is major concern and a major issue for understanding remissions. There are enough data available to make an educated guess that with poorly controlled diabetes, the overall output of insulin from a given beta-cell mass can be reduced by perhaps 75% (6–8). This is a major issue but also a therapeutic opportunity because it means that insulin secretion can be dramatically improved when glucose levels are normalized either by insulin treatment or improvement of insulin sensitivity.

What happens in the earliest stages of progression to diabetes when beta-cell secretion cannot keep up with demand? Even with glucose levels staying very close to normal there is a reduction in glucose-induced first-phase insulin release (FPIR) (9, 10). This striking change in secretion is frequently found shortly before the development of full-blown diabetes and can be seen as a “danger signal” (11). There has been a great deal of discussion about how or whether this can be blamed on elevations of glucose levels that are difficult to measure. The concept of “overwork” in the absence of hyperglycemia remains an ill-defined possibility (3). Some have argued that that the loss of FPIR is due not to overwork (or glucotoxicity) but to depletion of the readily releasable pool (RRP) of vesicles by high basal insulin secretion (12, 13). The loss of FPIR allows glucose to subsequently rise to levels where glucotoxicity comes into play and causes rapid deterioration.

For diabetes remissions we should focus on how much beta-cell mass is needed to maintain glucose control. A loss of 50% of mass is a key number because studies of partial pancreatectomy in humans indicate that this degree of deficiency leads to great risk of diabetes (10, 11). Moreover, studies of beta-cell mass in cadavers of people with T2D typically find a reduction in the range of 50% compared to non-diabetic controls (14, 15).

## As glucose levels climb the risk of accelerated beta-cell dysfunction increases

Although not widely appreciated, the trajectory of glycemic deterioration accelerates as impaired glucose tolerance advances to frank diabetes. This change in trajectory was observed in our animal studies (16) and then confirmed by the large Whitehall ll study of T2D (17). Thus, the time-period prior to diagnosis, which often includes impaired glucose tolerance, can be considered a time of glycemic stability, but as glucose levels rise the trajectory of glucose increases markedly. What this means in a practical sense is that in progression to both T1D and T2D, glucose levels climb slowly during a stable period but are at increased risk of suddenly shooting up too much higher levels, which are more difficult to treat. This is an important concept when the treatment goal is remission or partial remission. As is the case with the normal evolution of T2D, as glucose intolerance develops there is increased risk of change of the glucose trajectory to more unstable difficult-to-control diabetes. These differences also suggest that an intermediate treatment goal that can be considered a partial remission can be impaired glucose tolerance.

## Complete remissions are the goal, but partial remissions can also provide important benefit

The possibility of inducing remission of diabetes is receiving increasing attention, but as emphasized by others, its benefits are limited, and it is not a cure (18). Most people with diabetes will not be able to obtain a full remission because their reduction in beta-cell mass has reached a critical point of insufficiency, and for those who do, it will likely be limited in duration. However, there are benefits from obtaining a full remission for whatever duration is possible. Moreover, it may be that a partial remission can be helpful for a larger number of people in providing lowered risk of diabetes complications. Thus, IGT is a state that requires continued vigilance and treatment with drugs or diet and exercise to avoid relapse.

## Timing of the onset and reversal of glucose toxicity

We have limited information about this important question. We know that in rodents changes can start to occur within 24 hours (19, 20) and become more marked over the next several days. Reversal of glucose toxicity has not been thoroughly studied but from what we know from bariatric surgery glucose-induced FPIR is restored to at least near-normal in a few weeks (21).

## Progression of beta-cell dysfunction is similar for both T1D and T2D as indicated by remission with T1D and “Flatbush diabetes”

While the reasons for beta-cell damage in T1D and T2D are very different, the deterioration of function follows a very similar path. In both situations loss of glucose-induced FPIR is an early danger signal telling us that risk of the diabetes accelerating to an unstable pattern is increasing.

Both TID and what is called “Flatbush” (Flatbush refers to Brooklyn where the syndrome was described) diabetes can have impressive remissions and probably similar pathophysiology that we can learn from. For TID, it is not uncommon for there to be a “honeymoon”, a time of reasonable glycemic control without insulin or any other treatment for a few months after initiation of insulin treatment; this can be considered a remission. There was an impressive study in Brazil in which autoimmunity in T1D was aggressively treated with autologous stem cell transplants, and when partial or complete remission was obtained insulin levels were impressively increased (22). An equally impressive result was obtained with “Flatbush diabetes” (23). These were patients without autoimmune diabetes who had diabetic ketoacidosis and made a full recovery with no need for insulin or any other treatment. The best way to explain these cases is that beta-cell mass was partially maintained to about 50% of normal. We suspect that glucose toxicity caused major inhibition of insulin secretion and that activation of the sympathetic nervous system with severe illness provided further beta-cell inhibition and enhancement of hyperglycemia and ketoacidosis.

## Remissions are unlikely to restore islets to their formerly healthy state

Remissions can be very impressive as shown by truly normal glucose levels even with glucose challenges. The clear return of glucose-induced FPIR is also unequivocal. However, it seems very unlikely that the return is perfect. Now we have the tools to answer this important question. There may be permanent damage that limits the duration of a complete remission or the potential benefit of a partial remission.

## Beta-cells change with age

Beta-cells after remission are likely to be older having been exposed to prediabetes and diabetes stresses for considerable periods of time. Various treatment programs can lead to normal glucose levels as determined by HbA1c levels and continuous glucose monitoring, as well as return of glucose-induced FPIR and insulin sensitivity to the normal range. However, the hope that the islets would be indistinguishable from before seems unlikely. The number of senescent beta-cells increases with age, insulin resistance and the presence of T2D (24–26). Senescent cells are in growth arrest but continue to survive and function, although a number of important beta-cell genes have reduced expression, which suggest an impairment of insulin secretion (27). While senescence is thought to be a permanent state, more evidence suggest that it involves a cascade of changes over time (28) and that at early stages are partially reversible (27). They also express the senescence-associated secretory profile (SASP), which means they secrete soluble and insoluble factors that can cause adverse effects in surrounding tissue through paracrine interaction that can convert more cells to the senescent state. There is great current interest in the development of senolytic and senomorphic drugs (27) that can either delete the senescent cells or inhibit the SASP production.

Over the past few years there have been extraordinary advances in measuring beta-cell heterogeneity. New tools, including single-cell RNA sequencing, new imaging methods and studies of secretory function have provided a flood of new information. Recent studies of beta-cell aging using ^15^N mapping indicate that some beta-cells are able to divide a few times or perhaps not at all (29). We also know that with age human beta-cells accumulate lipofuscin, which can be found in a majority of adult beta cells (30) and serve as a marker for aging. Recent data also indicate that with age there is increased activation of ER stress and autophagy pathways (31). A key question is whether young beta-cells function better than old beta-cells. Islet transplant programs report that islets from old organ donors function less well than those from younger donors (32).

## Changes occurring with T2D

A separate issue concerns the changes that occur with T2D. We know that the phenotype of beta-cells exposed to hyperglycemia for even a short period of time changes dramatically (33). As an example of the magnitude of change, we looked at gene expression of rat islets exposed to mild hyperglycemia following partial pancreatectomy and found that 7844 genes of a total of 15,207 (52%) were differentially expressed (34). Our understanding of human T2D has greatly advanced dramatically because more well-characterized samples have become available, and the techniques are more sophisticated. These include laser-capture microdissection (35) and single-cell transcriptomics (36). A key question is whether these many changes come back to normal with remission and whether duration of time with hyperglycemia makes a difference. We know that impressive return of secretory function occurs with remission, but its longevity may be curtailed if genetic variants persist. With T2D, deterioration with time is thought to be more related to beta-cell failure than to insulin resistance (37). The likelihood that this is due to glucose toxicity provides even more reason to attempt to lower glucose levels.

## Bariatric surgery has been a guide to obtaining remissions for T2D

The success and popularity of bariatric surgery has provided us with an enormous amount of information about remissions of T2D. A study by Purnell and colleagues from 10 US hospitals reported on a 7-year follow-up after Roux-en-Y bypass (RYGB) and laparoscopic gastric banding (LAGB) (1). Of the 2256 participants, 827 had known diabetes. Remissions were described as being either complete or partial.

Two years after surgery, for the RYGB group, 51.3% (261/509) had a complete remission. For the LAGB group, 22.6% (35/155) had a complete remission. As expected, those obtaining remission in both groups had shorter duration of diabetes, better baseline HbA1c values and were taking fewer diabetes medications. These results are similar to those reported by others. The results from LAGB tell us what can be expected from just weight loss. The mechanisms that account for the better results from RYGB than with LAGB remain ill-defined (38).

## Contributions of weight loss and glucose control to remissions

When the benefits of bariatric surgery glucose control are reported, weight loss is often described as a key determent of success (39). However, it can be argued that the key driver is reduction of glucose that results in amelioration of glucose toxicity. It is clear that impressive effects on glucose control are often seen in the early stages of a treatment program before significant weight loss has occurred.

## Medical approaches to obtain remissions

The idea that lowering glucose levels might lead to remission of diabetes control was tested and reported on in 1997 by the group of Cerasi (40) with the finding that transient intensive insulin therapy given for a period of 2 weeks could result in improved glycemic control lasting for as long as several months. This was followed by a report from Wang and co-workers (41) who used intensive insulin therapy on 382 individuals with newly diagnosed T2D, finding that improved control could last for many months. Retnakaran et al. (42) gave 4-8 weeks of intensive insulin therapy to 34 patients with established T2D and found improved fasting glucose levels for 68% of the subjects.

As interest in remissions increases, there is greater appreciation that the number of patients likely to obtain such benefit is a small proportion of the T2D population. It is also becoming apparent that the most important intervention is weight loss and that exercise without wight loss has less obvious value (18, 43, 44).

The surgical results described above provide important insights about what might be accomplished when remission is attempted with medical treatment. The superior results with RYGB are thought to be partly due to weight loss, but the mechanisms responsible for the other benefits are still not understood (38). The LABG results help us understand what weight loss can accomplish, but medical treatment can use more interventions so it might be possible to obtain better results. There is great interest in the long-acting drugs that act as agonists for both glucagon-like peptide 1 (GLP-1) and glucagon inhibitory peptide (GIP) receptors. Such drugs as long-acting drug tirzepatide (45) cause weight loss and stimulation of insulin secretion. This approach and related therapies can be expected to provide more complete and partial remissions.

## Summary

There has been impressive progress in the past decade in understanding the pathogenesis of both T1D and T2D as it relates to the importance of beta-cell insufficiency. Glucose toxicity exerts profound inhibition of insulin secretion. The realization that this is reversible, and the development of new therapeutic strategies means that both complete and partial remissions can become more prominent goals of treatment.

## Author contributions

All authors listed have made a substantial, direct, and intellectual contribution to the work and approved it for publication.
